# Supplementary material for: Highlighting the Major Role of Cyclin C in Cyclin-Dependent Kinase 8 Activity through Molecular Dynamics Simulations
Source: Int J Mol Sci. 2024 May 15;25(10):5411. doi: 10.3390/ijms25105411 (PMC11121562; doi:10.3390/ijms25105411)
Supplement: Supplementary file 1 [file ijms-25-05411-s001.zip › ijms-2958960-supplementary.pdf]

SUPPORTING INFORMATION

Section S1: Model building and system preparation.

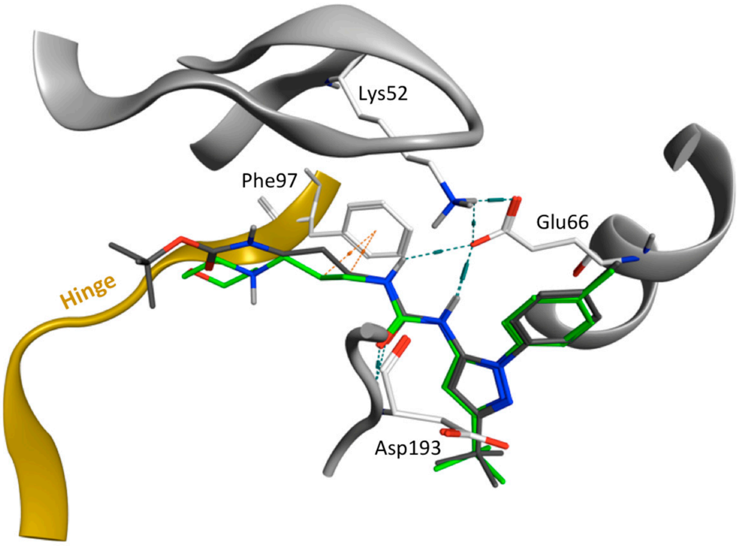

Figure S1: Binding site of the crystal structure of human CDK8 in complex with inhibitors 0SR and 0SO from, respectively, PDB ID 4F6U and 4F7L.

Inhibitors 0SR to 0SO are colored, respectively, in green and black. Interactions between the urea and the common scaffold of the inhibitors and the residues Glu66 and Asp173 are shown in blue dashed lines. The protein is represented by the white cartoon, except the hinge, which is the colored yellow cartoon.

```
Uniprot_Cyclin-C      ---MAGNFWQSSHYLQWILDKQDLLKERQKDLKFLSEEEYWKLQIFFTNVIQALGEHLKL
4F6U.B                DKAMAGNFWQSSHYLQWILDKQDLLKERQKDLKFLSEEEYWKLQIFFTNVIQALGEHLKL
                         *********************************************************

Uniprot_Cyclin-C      RQQVIATATVYFKRFYARYSLKSIDPVLMAPTCVFLASKVEEFGVVSNTRLIAAATSVLK
4F6U.B                RQQVIATATVYFKRFYARYSLKSIDPVLMAPTCVFLASKVEEFGVVSNTRLIAAATSVLK
                      ************************************************************

Uniprot_Cyclin-C      TRFSYAFPKEFPYRMNHILECEFYLLELMDCCLIVYHPYRPLLQYVQDMGQEDMLLPLAW
4F6U.B                TRFSYAFPKEFPYRMNHILECEFYLLELMDCCLIVYHPYRPLLQYVQDMGQEDMLLPLAW
                      ************************************************************

Uniprot_Cyclin-C      RIVNDTYRTDLCLLYPPFMIALACLHVACVVQQKDARQWFAELSVDMEKILEIIRVILKL
4F6U.B                RIVNDTYRTDLCLLYPPFMIALACLHVACVVQQKDARQWFAELSVDMEKILEIIRVILKL
                      ************************************************************

Uniprot_Cyclin-C      YEQWKNFDERKEMATILSKMPKPKPPPNSEGEQGPNGSQNSSYSQS
4F6U.B                YEQWKNFDERKEMATILSKMPKPKPPP-------------------
                      ***************************
```

Figure S2: Alignment of the UNIPROT sequence of the human Cyclin C with the Cyclin C sequence of 4F6U (PDB ID).

```
Uniprot_CDK8     --MDYDFKVKLSSERERVEDLFEYEGCKVGRGTYGHVYKAKR-KDGKDDKDYALKQIE--
4F6U.A           DKMDYDFKVKLSSERERVEDLFEYEGCKVGRGTYGHVYKAKR-KDGKDDKDYALKQIE--
1BI8.A           --MEKDGLCRADQQYECVAE--------IGEGAYGKVFKARDLKNG--GRFVALKRVRVQ
1G3N.A           --MEKDGLCRADQQYECVAE--------IGEGAYGKVFKARDLKNG--GRFVALKRVRVQ
                   *: *   : ..: * * :        :*.*:**:*:**:  *:*  .:  ***::.

Uniprot_CDK8     -GT-GISMSACREIALLRELK---HPNVISLQKVF-LSHADR--KVWLLFDYAEHDLWHI
4F6U.A           -GT-GISMSACREIALLRELK---HPNVISLQKVF-LSHADR--KVWLLFDYAEHDLWHI
1BI8.A           TGEEGMPLSTIREVAVLRHLETFEHPNVVRLFDVCTVSRTDRETKLTLVFEHVDQDLTTY
1G3N.A           TGEEGMPLSTIREVAVLRHLETFEHPNVVRLFDVCTVSRTDRETKLTLVFEHVDQDLTTY
                  *  *:.:*: **:*:**.*:   ****: * .*  :*::**  *: *:*::.::**

Uniprot_CDK8     IKFHRASKANKKPVQLPRGMVKSLLYQILDGIHYLHANWVLHRDLKPANILVMGEGPERG
4F6U.A           IKFHRASK-----VQLPRGMVKSLLYQILDGIHYLHANWVLHRDLKPANILVMGEGPERG
1BI8.A           L-----DKVPEPGV--PTETIKDMMFQLLRGLDFLHSHRVVHRDLKPQNILVTSSG----
1G3N.A           L-----DKVPEPGV--PTETIKDMMFQLLRGLDFLHSHRVVHRDLKPQNILVTSSG----
                 :     .*     *  *   :*.:::*:* *:.:**:: *:****** ****  ..*

Uniprot_CDK8     RVKIADMGFARLFNSPLKPLADLDPVVVTFWYRAPELLLGARHYTKAIDIWAIGCIFAEL
4F6U.A           RVKIADMGF-----------------VVTFWYRAPELLLGARHYTKAIDIWAIGCIFAEL
1BI8.A           QIKLADFGLARIYSFQMA----LTSVVVTLWYRAPEVLLQSS-YATPVDLWSVGCIFAEM
1G3N.A           QIKLADFGLARIYSFQMA----LTSVVVTLWYRAPEVLLQSS-YATPVDLWSVGCIFAEM
                 ::*:**:*:                 ***:******:** :  *:..:*:*::******:

Uniprot_CDK8     LTSEPIFHCRQEDIKTSNPYHHDQLDRIFNVMGFPADKDWEDIKKMPEHSTLMKDFRRNT
4F6U.A           LTSEPIFHCRQE-----NPYHHDQLDRIFNVMGFPADKDWEDIKKMPEHSTLMKDFRRNT
1BI8.A           FRRKPLFR-GSSDV--------DQLGKILDVIGLPGEEDWPRDVALPRQAFHSKSAQP--
1G3N.A           FRRKPLFR-GSSDV--------DQLGKILDVIGLPGEEDWPRDVALPRQAFHSKSAQP--
                 :  :*:*:  ..          ***.:*::*:*:*.::**     :*.::   *. :

Uniprot_CDK8     YTNCSLIKYMEKHKVKPDSKAFHLLQKLLTMDPIKRITSEQAMQDPYFLEDPLPTSDVFA
4F6U.A           YTNCSLIKYMEKHKVKPDSKAFHLLQKLLTMDPIKRITSEQAMQDPYFLEDPLPTSDVFA
1BI8.A           ---------IEKFVTDIDELGKDLLLKCLTFNPAKRISAYSALSHPYFQDLERCKENLDS
1G3N.A           ---------IEKFVTDIDELGKDLLLKCLTFNPAKRISAYSALSHPYFQDLERCKENLDS
                          :**. .. *. . .** * **::* ***:: .*:..*** :    ..:: :

Uniprot_CDK8     GCQIPYPKREFLTEEEPDDKGDKKNQQQQQGNNHTNGTGHPGNQDSSHTQGPPLKKVRVV
4F6U.A           GCQIPYPKREFLTEEEPDDKGDKKNQQQQQGNNHTNGTGHPGNQDSSHTQGPPLKK----
1BI8.A           ------------------------------------HLPPSQNTSELNTA--------
1G3N.A           ------------------------------------HLPPSQNTSELNTA--------
                                                         * .*::*. : .
```

Figure S3: Alignment of the human sequences of CDK8 (canonical UNIPROT sequence and 4F6U (PDB ID) sequence) with the template sequences of human CDK6 (1BI8 and 1G3N (PDB ID)).

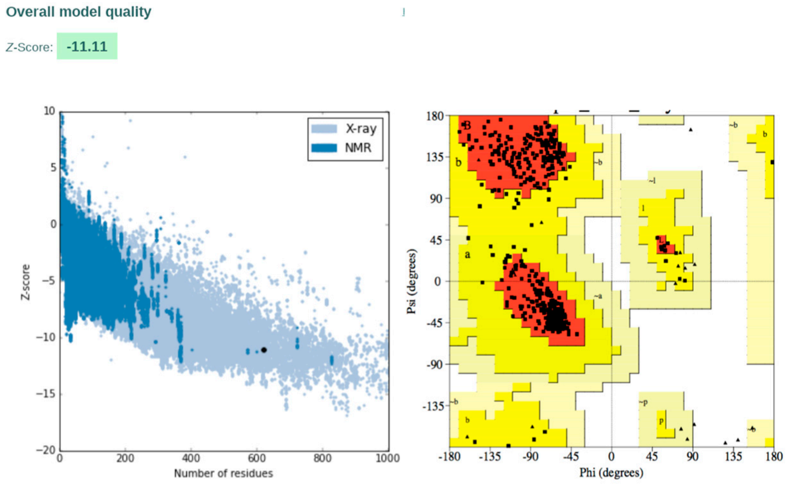

Figure S4: Model validation.
(**Left**) Plot of the Z-score. The *Z*-score indicates the overall model quality. The plot contains the *Z*-scores of all experimentally determined protein chains in the current PDB. In this plot, groups of structures from different sources (X-ray, NMR) are distinguished by different colors. The Z-score of the reconstructed model of 4F6U (*PDB id.*) is represented by a black point. Its value is within the range of scores typically found for native proteins of similar sizes. **(Right)** Ramachandran plot showing that most of the model residues display backbone dihedral angle phi and psi values in the energetically allowed regions.

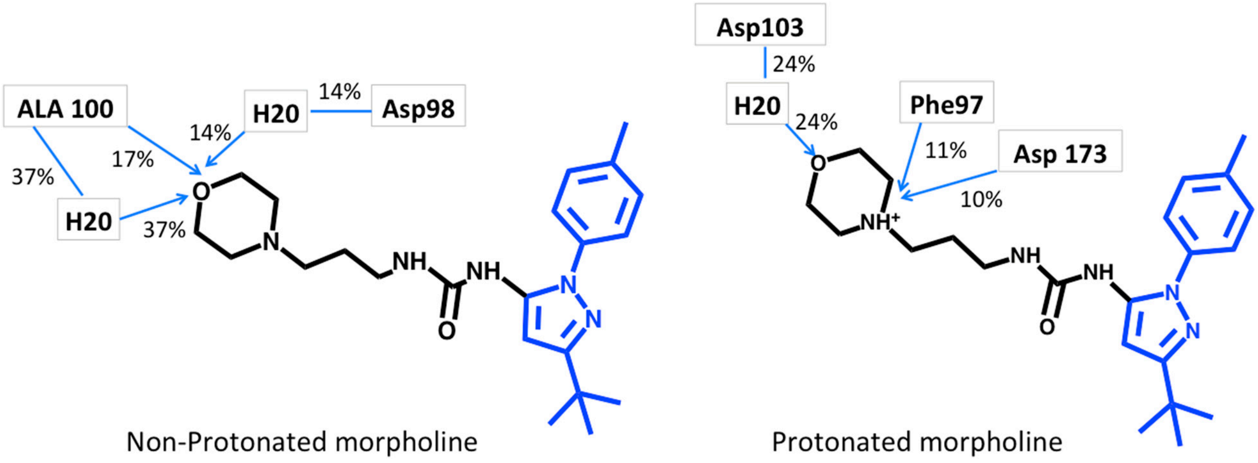

Figure S5: Difference in protein–ligand interaction between the non-protonated and protonated forms of the morpholine of inhibitor 0SR.
The interactions involving the scaffold (drawn in blue) and the urea are not represented because no difference has been noted. These interactions have been calculated using a brute-force MD simulation of 1 microsecond. The percentage represents the duration of the simulation during which the interaction is maintained. With the protonated morpholine, the interaction with Ala100 is not observed any longer.

Section S2: Effect of the exclusion of CycC on the structure and dynamics of CDK8.

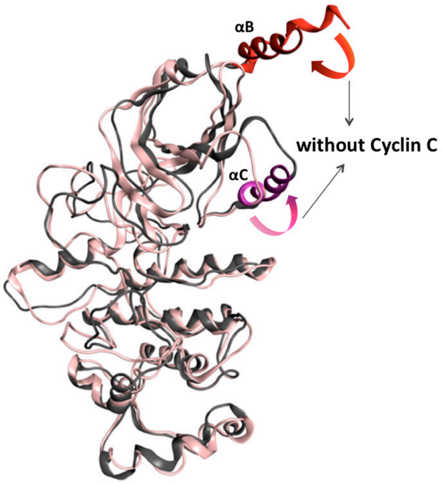

Figure S6: Conformational changes observed in the absence of CycC.
The protein is represented by a gray ribbon in system 2a (without CycC) and in light pink in system 1a (with CycC), except the αB and αC helix.

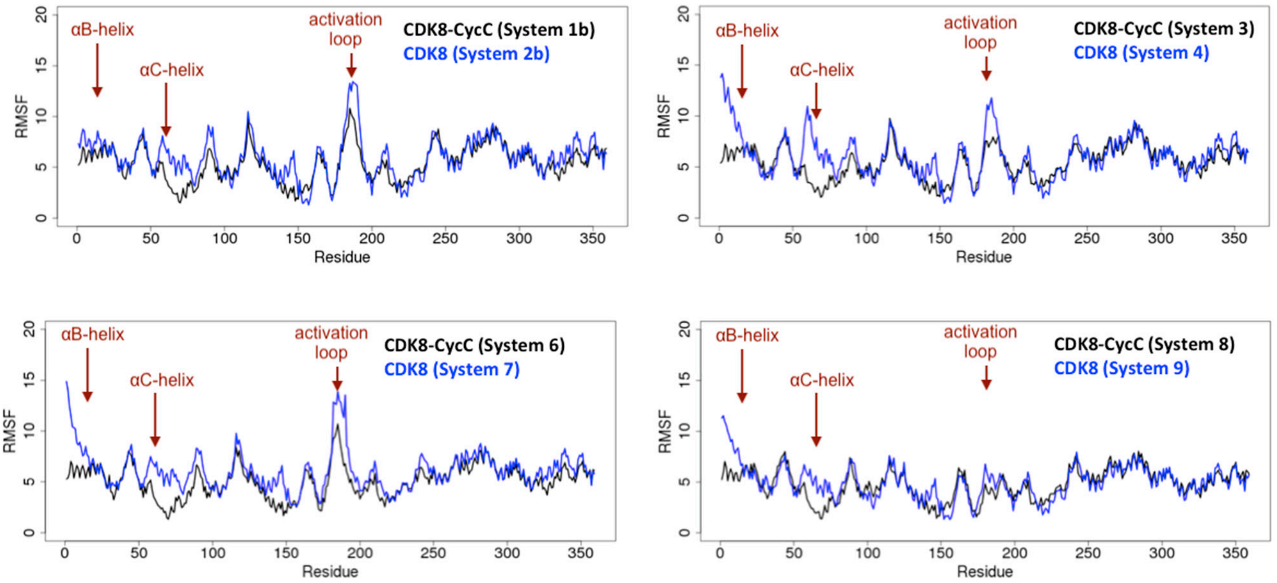

Figure S7: Comparison of the root mean square atomic fluctuations of CDK8 in the presence/absence of CycC.

Besides the application of PCA to a combined trajectory (with/without CycC), PCA was also applied to each individual trajectory. The goal is to capture the major CDK8 motions observed in the different systems and to assess if the presence of the CycC impacts the dynamics of CDK8. To visualize the largest amplitude motions, a PDB-format trajectory has been produced that interpolates between the most dissimilar structures in the distribution along PC1.

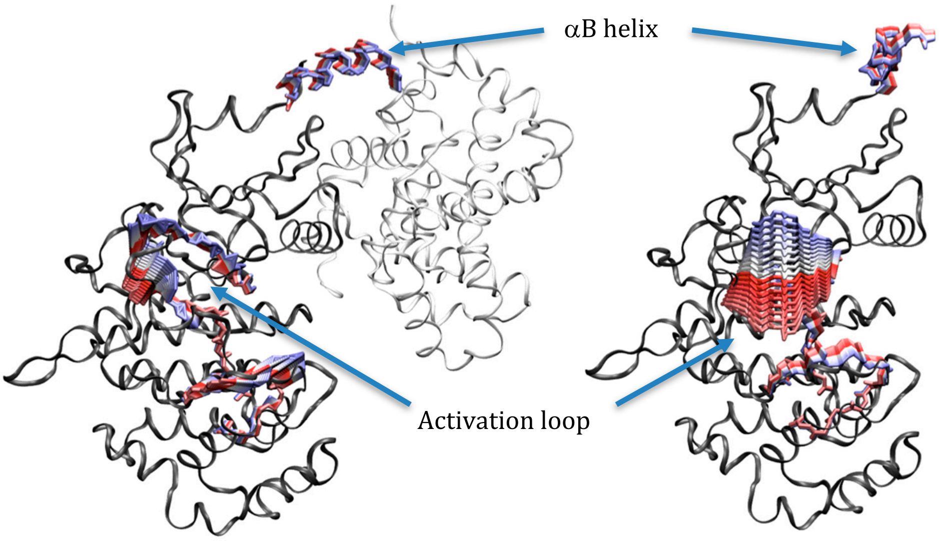

Figure S8: Largest fluctuation motions (PC1) in the *DMG-out* conformation complex in the presence (left) and absence (right) of CycC.
The regions with the largest moves are represented by a color gradient from blue to red to capture the dynamics.

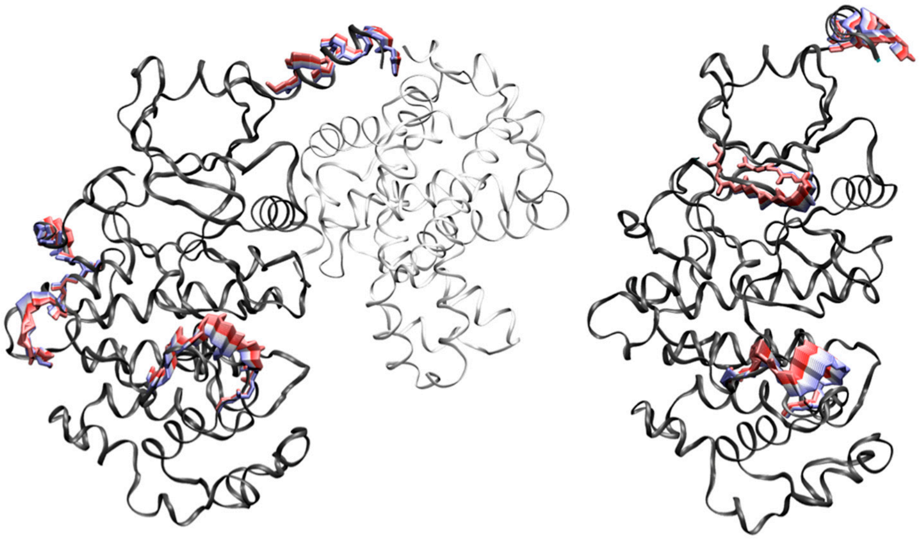

Figure S9: Largest fluctuation motions (PC1) in the *DMG-in* conformation complex in the presence (left) and absence (right) of CycC.
The regions with the largest moves are represented by a color gradient from blue to red to capture the dynamics.

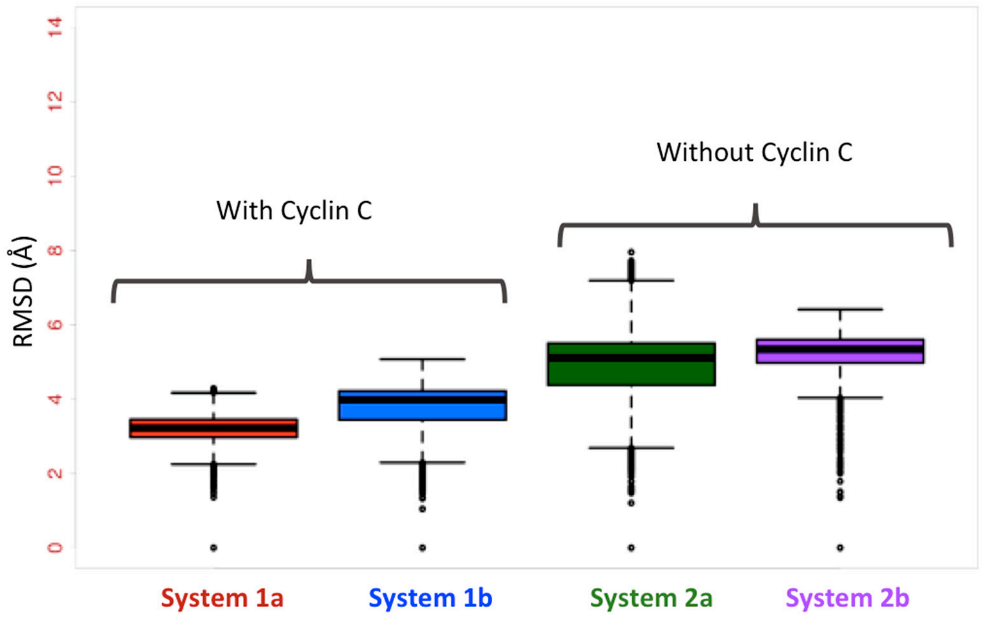

Figure S10: Distribution of the ligand RMSD in the presence and absence of CycC.

Section S3: Characterization of the protein–protein interactions between CDK8 and CycC.

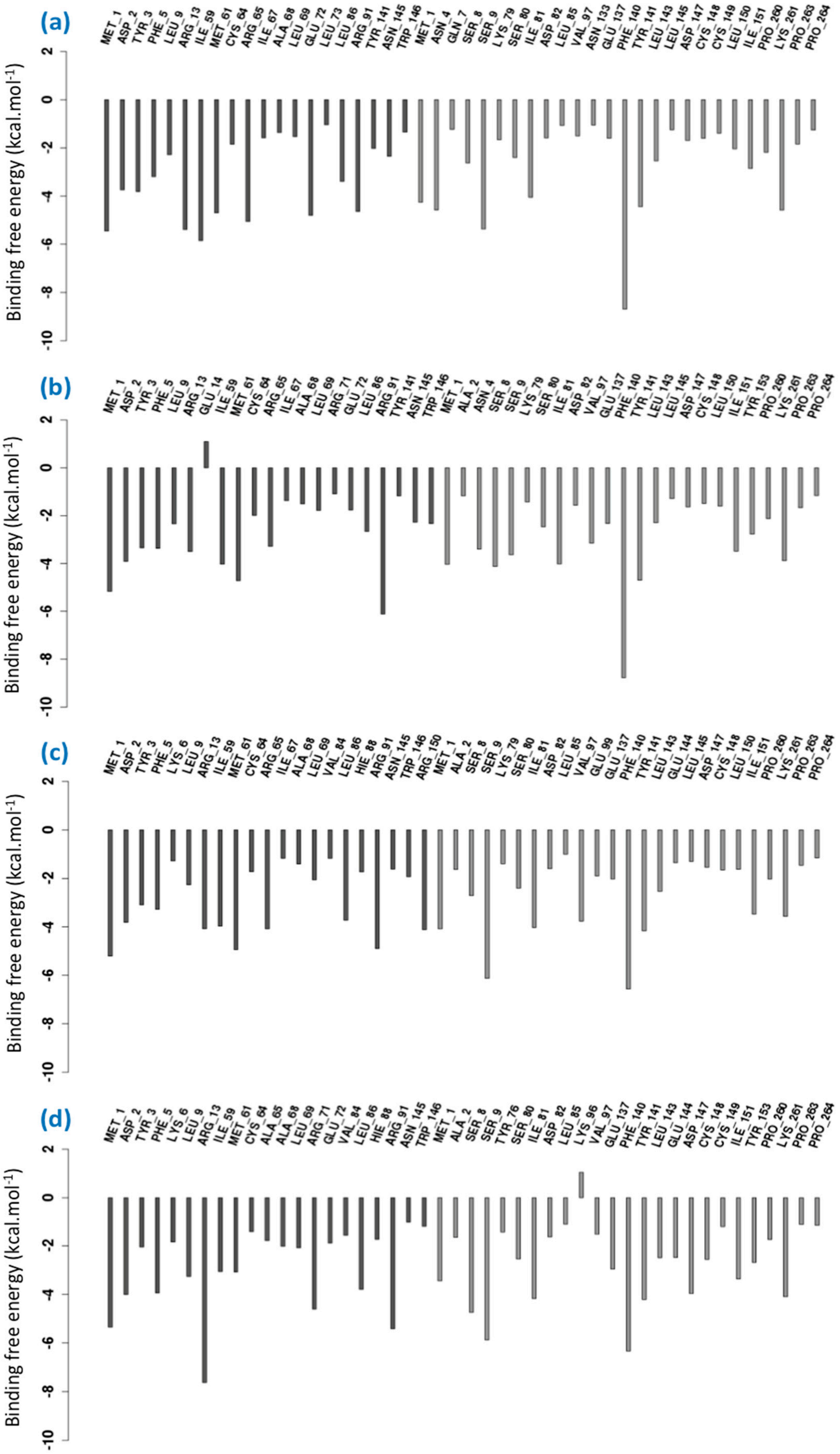
(a)
(b)
(c)
(d)
Binding free energy (kcal.mol-1)

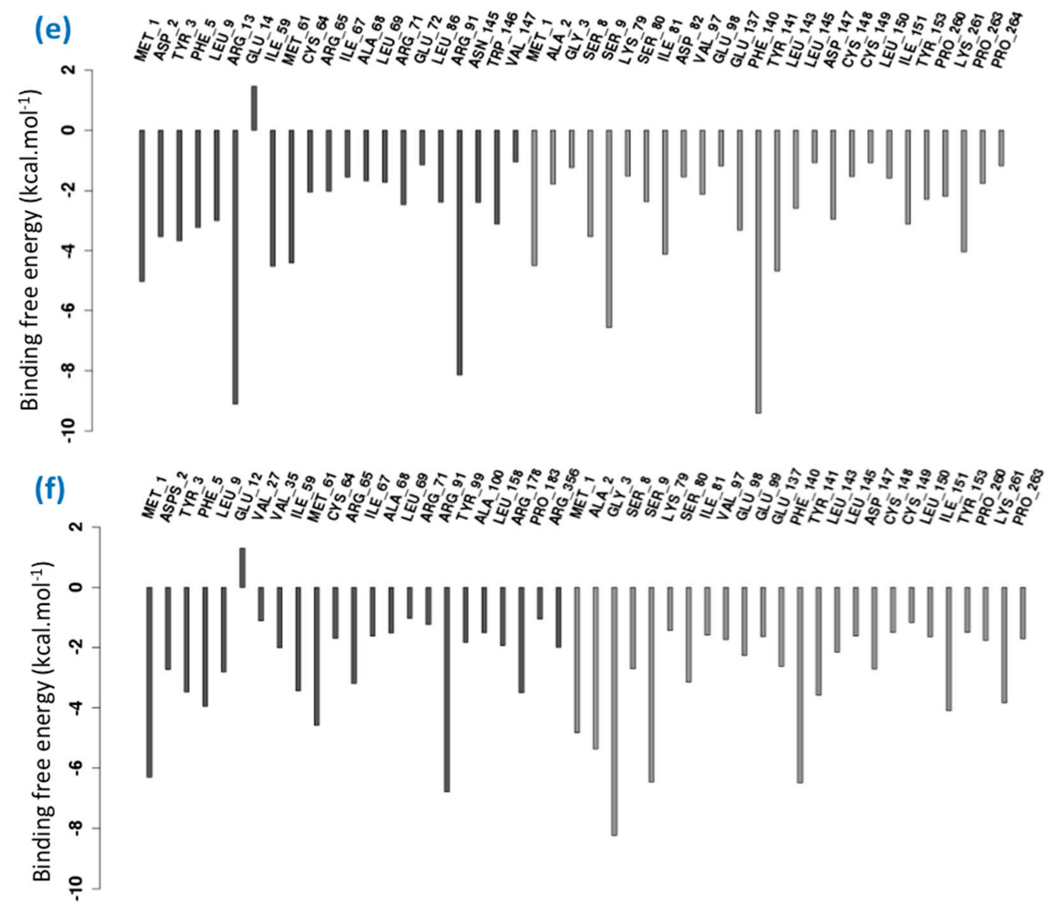

Figure S11: Energy contribution of important residues of each studied CDK8–CycC complex.

An important residue is defined as a residue in which the absolute value of ΔG_{total} is superior to 1kcal.mol^{-1}. (**a**): System 1a, (**b**): system 1b, (**c**): system 3, (**d**): system 5, (**e**): system 6, (**f**): system 8. Residues whose bars are colored in dark gray belong to CDK8, and those in light gray belong to CycC.

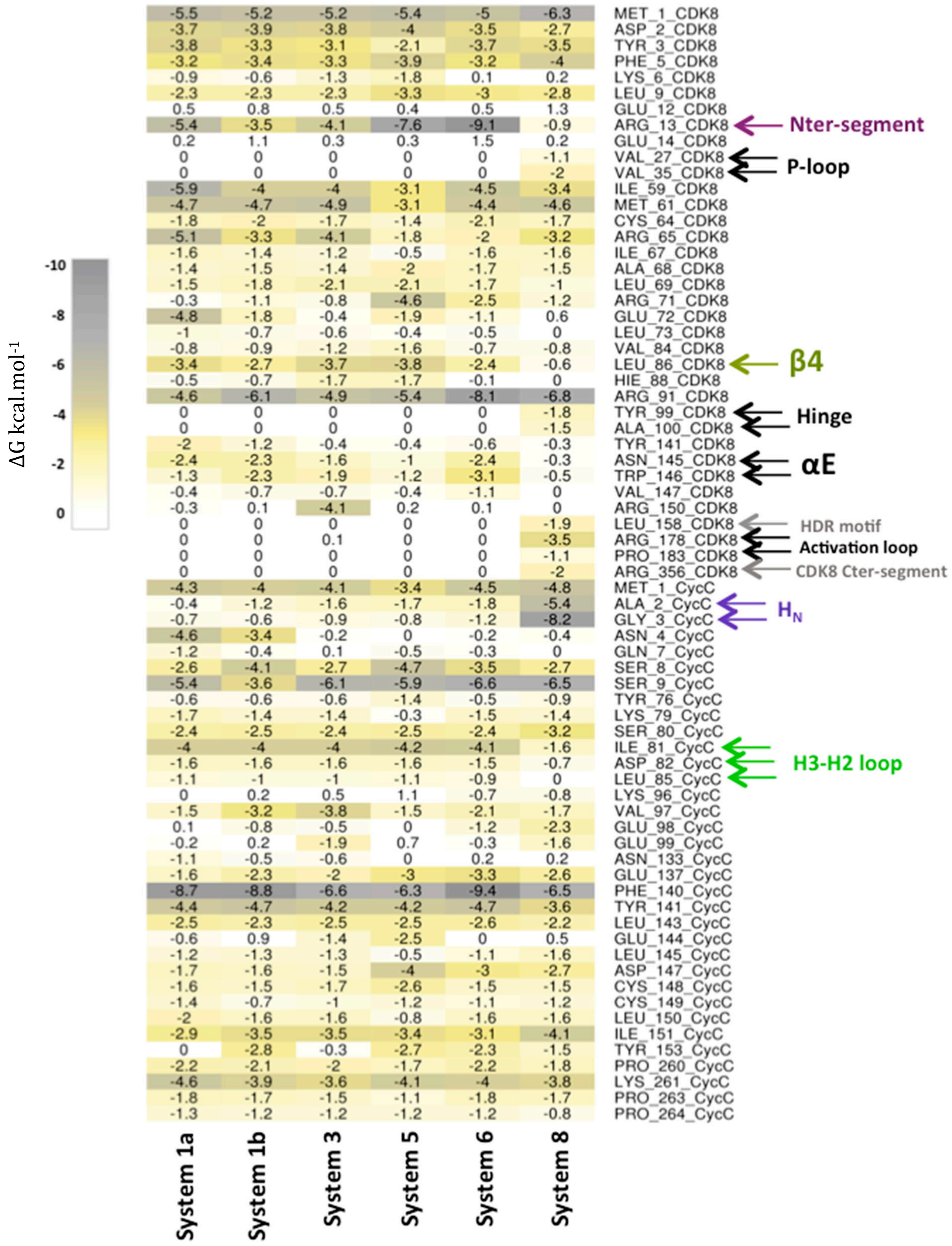

Figure S12: Matrix of the per-residue energy contribution (ΔG without entropy in kcal.mol^{-1}) of the residues that present at least one significant energy contribution (absolute(ΔG) > 1 kcal.mol^{-1}) in one of the studied CDK8–CycC complexes.
Arrows point to residues that contribute differently to the *DMG-in* and *DMG-out* conformations. The secondary structure they belong to is also indicated and colored following the same color code as Figure 5 (gray color is used to distinguish two narrow indications).

Section S4: Activation mechanism of CDK8.

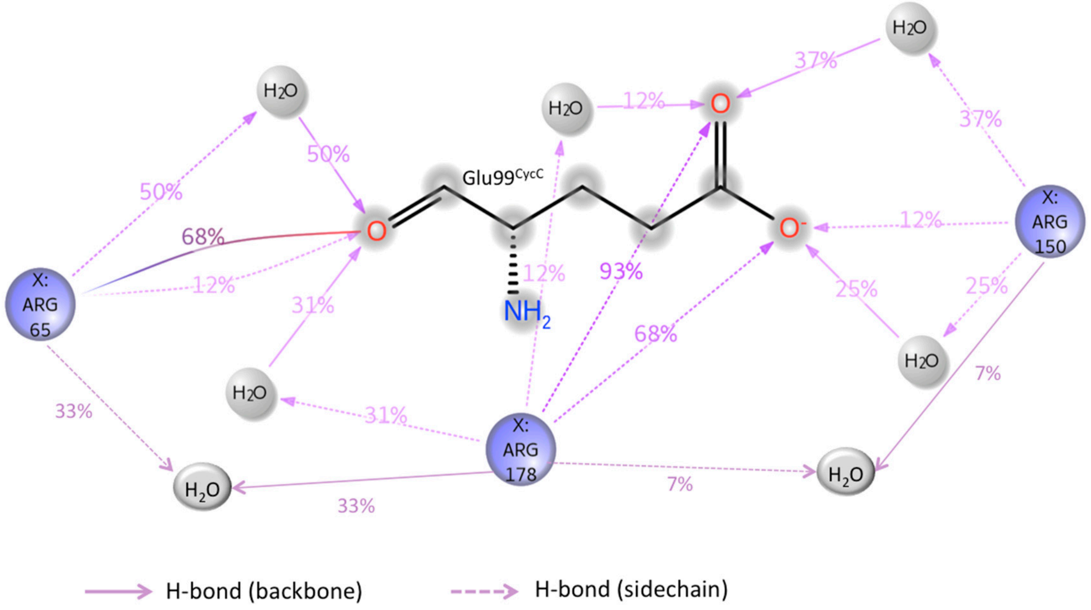

Figure S13: Interaction network between the three conserved arginines of CDK8 (Arg65^{CDK8}, Arg150^{CDK8}, and Arg178^{CDK8}) and the glutamate 99 of the CycC in the *DMG-in* conformation system.
The interactions were calculated using a 1 μs simulation of system 8.

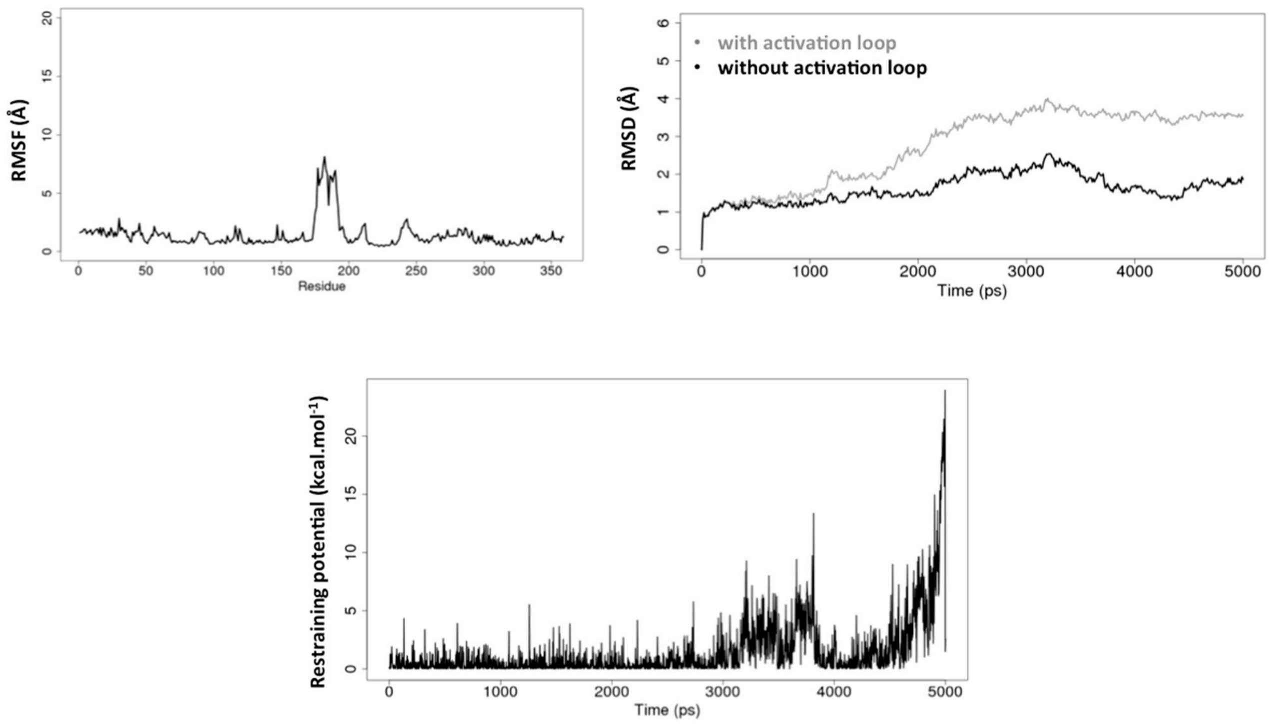

Figure S14: Analysis of the stability of the protein structure over time during the TMD simulation through the investigation of the RMSF, RMSD, and the restraining potential ($V_{restraint}$).

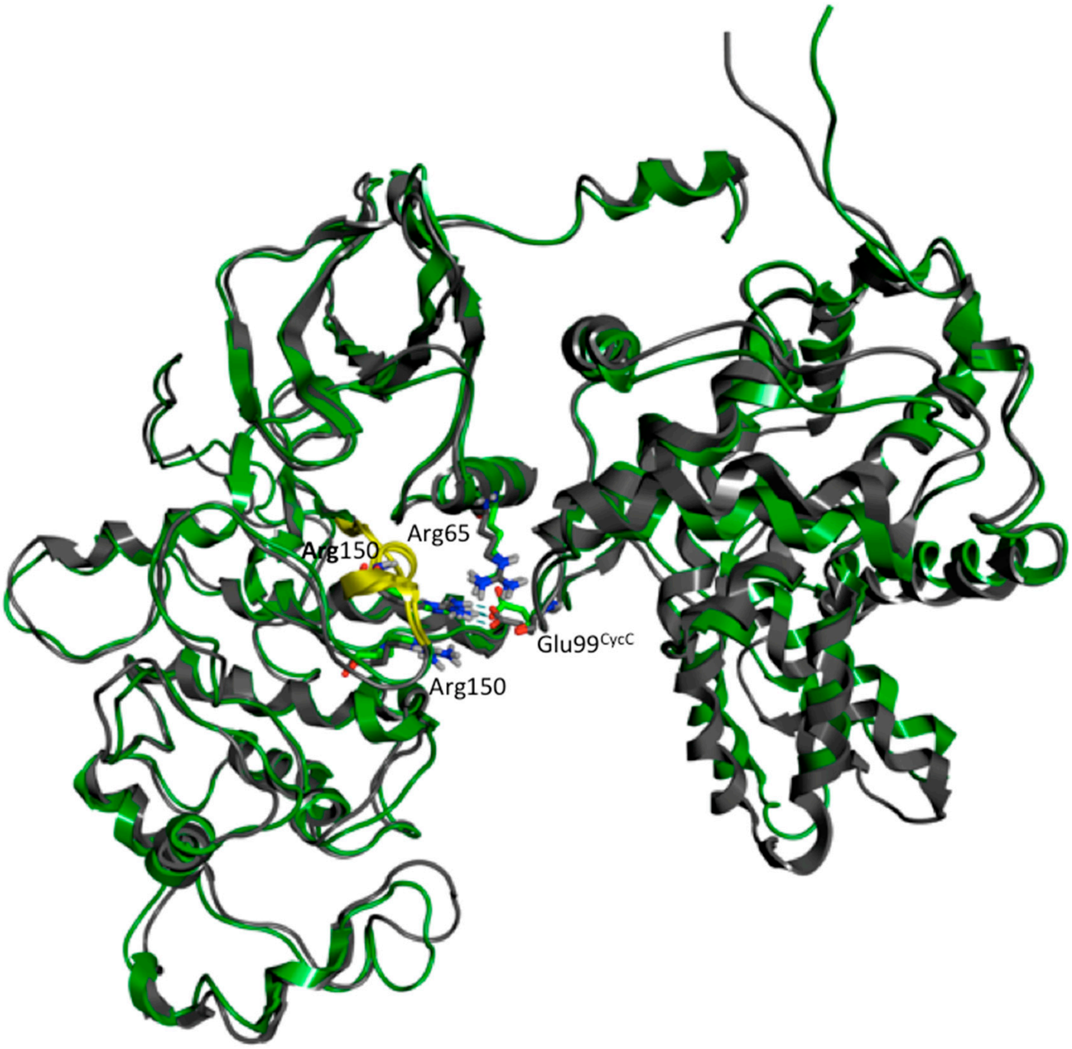

Figure S15: Comparison of the structures of CDK8–CycC in the *DMG-in* conformation obtained from TMD and cMD simulations.
The structure obtained from 5 ns of TMD simulation followed by 50 ns of cMD (in green ribbon) was compared to the structure obtained from the 1 µs cMD simulation (in dark gray ribbon). Residues 171 to 182 of the CDK8 protein are colored in yellow. These are the residues that were constrained during the TMD simulation. The three conserved arginines (Arg65^{CDK8}, Arg150^{CDK8}, and Arg 178^{CDK8}) are represented as sticks.
